# Evaluating the Impacts of Community-Campus Engagement on Population Health in Ottawa and Thunder Bay, Canada: Protocol for a Mixed Methods Contribution Analysis

**DOI:** 10.2196/58546

**Published:** 2025-01-17

**Authors:** David Buetti, Cynthia Larche, Michael Fitzgerald, Isabelle Bourgeois, Erin Cameron, Kady Carr, Tim Aubry, Sydney Persaud, Claire E Kendall

**Affiliations:** 1 Department of Health Management, Evaluation, and Policy (DGEPS) School of Public Health (ESPUM) University of Montreal (UdeM) Montreal, QC Canada; 2 Northern Ontario School of Medicine Sudbury, ON Canada; 3 Bruyère Health Research Institute Ottawa, ON Canada; 4 Faculty of Education University of Ottawa Ottawa, ON Canada; 5 Ottawa Neighbourhood Study University of Ottawa Ottawa, ON Canada; 6 Faculty of Social Sciences University of Ottawa Ottawa, ON Canada; 7 Faculty of Medicine University of Ottawa Ottawa, ON Canada

**Keywords:** community-campus engagement, population health, contribution analysis, mixed methods, health determinants, community health, CityStudio, theory of change, impact evaluation

## Abstract

**Background:**

Municipalities play a crucial role in population health due to their community connections and influence on health determinants. Community-campus engagement (CCE), that is, collaboration between academic institutions and communities, is a promising approach to addressing community health priorities. However, evidence of CCE’s impact on population health remains limited. Measuring the impacts of CCE is inherently complex due to factors such as diverse stakeholders, context-specific variables, and dynamic interactions within a community.

**Objective:**

This study aims to develop robust evidence on the impacts of CCE on population health outcomes in Ottawa and Thunder Bay, Ontario, Canada, focusing on 5 shared health priorities: housing, discrimination, poverty, violence, and mental health.

**Methods:**

We will use a proven CCE model called CityStudio, which has been implemented in both cities. We will use Mayne's mixed methods contribution analysis in three stages: (1) formulating a theory of change that outlines the expected contributions of CCE to population health outcomes; (2) gathering qualitative and quantitative data in line with the established Theory of Change; the data will be collected from various sources, including case studies of existing CityStudio projects, a web-based CCE stakeholder survey, a literature review, and population and community health data; and (3) reviewing the gathered evidence to determine the extent of CCE impacts on population health.

**Results:**

Ethical approval for this project was granted in May 2023. We have since initiated stage 1 by reviewing the literature to inform the development of the theory of change. We expect to complete this study by May 2026.

**Conclusions:**

This study will address two critical gaps about how improving health outcomes depends on CCE: (1) how academic institutions can best engage with their communities to improve population health outcomes, and (2) how municipalities can engage with academic institutions to address their community health priorities. Conducting our work in differing contexts will allow us to consider a broader range of other influences on outcomes, thus making our work applicable to various settings and outcomes.

**International Registered Report Identifier (IRRID):**

PRR1-10.2196/58546

## Introduction

### Background

Achieving improved population health outcomes requires a comprehensive, multisectoral approach beyond healthcare [1-3]. With their robust community ties and influence over various health determinants, municipalities are well-positioned to drive progress [1-3]. In Canada, local governments and community partners often identify their communities’ priority health needs through established safety plans or social policy frameworks [3]. These needs are expressed in Ontario, the country’s most populous province, through provincially mandated Community Safety and Well-Being Plans (CSWBPs) [4]. Updated every 4 years, these plans serve as strategic roadmaps, guiding municipalities' efforts to improve population health outcomes [4,5].

Collaborations between municipalities and their local academic institutions have the potential to contribute positively to the health of the local community [6-9]. Through collaborative efforts, community-campus engagement (CCE) fosters mutually beneficial relationships between local stakeholders (eg, community organizations, city representatives, and residents) and academic institutions (eg, universities and research centers) [6-8]. Municipalities can contribute their deep understanding of local needs and priorities to such engagement, while academic institutions provide research expertise and resources [6,8,10]. This collaborative framework facilitates shared decision-making, efficient resource allocation, and the development of tailored strategies to directly address specific health concerns within the community, ultimately leading to improved health outcomes [6,10].

CCE can take various forms, such as community-based research and service learning [6,10]. Community-based research involves collaboration between faculty members, students, and local communities to address critical issues [6,10]. On the other hand, service-learning integrates classroom learning with community service, equipping students with the skills to tackle real-world challenges while also improving their communities [6,10]. Each form can potentially contribute to the community’s health in different ways. For instance, community-based research can identify emerging health disparities in specific neighborhoods, while service-learning programs can equip future health care professionals with the skills to address these issues.

While CCE shows promise in supporting municipalities’ health agenda goals, there remains significant uncertainty about the specific mechanisms and pathways through which it influences population health outcomes, underscoring the need for further research to understand its impact at the municipal level [11]. Existing literature in this area is mainly reflective [12,13] or qualitative [14,15], primarily focusing on health outcomes resulting from individual partnerships [16-19] and the perspectives of faculty and students involved in CCE [10,14,20,21]. Although these studies provide valuable insights, they offer only a partial understanding of CCE’s broader impact on population health because they do not fully investigate the pathways, conditions, and contextual factors that influence outcomes.

Some studies have reported positive health outcomes, such as reduced substance misuse [16] and increased physical activity [17]. However, these studies often fail to clearly outline the change processes that explain these positive outcomes, focusing mainly on end results. This emphasis on outcomes, with insufficient attention to the processes, limits our understanding of how specific actions, stakeholder interactions, and contextual factors contribute to health improvements. Consequently, the complexity of the mechanisms driving these changes remains underexplored, making it difficult to pinpoint the pathways through which CCE initiatives achieve their impact. This knowledge gap highlights the need for more robust research to evaluate the impact of CCE on population health at the municipal level that specifically focuses on the contexts and mechanisms that result in change.

### Contribution Analysis: a Promising Approach for Evaluating the CCE Impacts

As with community engagement in general, the complexity of measuring CCE impacts on population health makes experimental approaches to measuring CCE impacts neither practical nor feasible, as many factors cannot be controlled for [22], such as the involvement of diverse stakeholders with diverging interests (eg, local governments, faculty and students, community groups) [15,23,24], the evolving dynamics of operations [25], and the influence of external factors like governmental policies and local socioeconomic conditions [15,26,27]. Experimental approaches may thus overlook the underlying causes of an intervention’s success or failure, providing limited insights into the causal mechanisms at play [28,29].

Contribution analysis is a theory-based impact evaluation approach that is particularly suited to contexts of high uncertainty, where the goal is not to prove causation definitively but to reduce uncertainty by establishing a plausible association between interventions and outcomes [28-32]. This emphasis on reducing uncertainty makes CA particularly suitable for evaluating the CCE impact on population health [11].

An integral component of contribution analysis is the theory of change, which outlines the causal mechanisms in a results chain running from inputs to impact and illustrates how the intervention being examined is expected to bring about change [31]. However, the theory of change goes beyond a standard results chain or logic model by elucidating assumptions, risks, unintended effects, and other vital factors underpinning the relationships [30,31].

Narrative statements, known as contribution claims, are generated once the theory of change has been developed [32]. These claims are presented as hypotheses, proposing how the intervention’s activities and outputs contribute to the observed outcomes while considering influencing factors and context [30]. The theory of change and contribution claims are refined and validated iteratively using qualitative and quantitative data and stakeholder participation [22,32]. This process alternates between theory development and empirical testing, resulting in a robust, evidence-based theory of change [30]. The ensuing theory is the foundation for the contribution story, which offers a detailed account of the intervention’s impact and the stakeholders' perspectives [33].

Contribution analysis may be carried out at 3 levels: minimalist, direct influence, and indirect influence, each tailored to the complexity of the intervention and the depth of the assessment [22]. Minimalist contribution analysis is ideal for interventions with clear, measurable outcomes. It formulates a theory of change, confirms output delivery, and validates contribution claims using existing data and evidence [22,32]. Direct influence contribution analysis is suitable for complex interventions with diverse outcomes, as it builds on minimalist analysis by using empirical data to confirm the intervention’s contribution to the impact [22]. Indirect influence contribution analysis, suitable for complex interventions with uncertain, emergent outcomes, builds on direct influence analysis by further testing contribution claims against alternative explanations, offering a more comprehensive understanding of what caused an observed impact [31,32].

### Objective

This research project uses contribution analysis to rigorously assess and reduce uncertainty about whether, how, and to what extent CCE has contributed to improving population health in Ottawa and Thunder Bay, Ontario, Canada. The study will focus on 5 specific shared population health priorities for both municipalities: housing, discrimination, poverty, violence, and mental health. Our specific objectives are as follows.

- Identify and outline the key mechanisms and processes through which CCE is anticipated to impact population health outcomes in Ottawa and Thunder Bay.
- Assess the extent to which CCE has contributed to the targeted population health outcomes, evaluating their impact against the mapped mechanisms and processes.

## Methods

### Study Setting

Our study will be conducted in Thunder Bay and Ottawa, 2 Ontario municipalities with medical schools and health education institutions. These cities have developed CSWBPs by reviewing local data comprehensively and holding community consultation sessions to identify their population’s unique needs and characteristics [34,35]. Despite differences between the Ottawa and Thunder Bay CSWBPs, both cities have identified 5 similar priorities related to population health, as outlined in Table 1.

**Table 1:** Shared priorities in the Thunder Bay and Ottawa Community Safety and Well-Being Plans.

Priority areas	Description
Housing	Ensuring all residents have access to safe, affordable, and suitable housing.
Discrimination	Addressing systemic issues perpetuating discrimination, marginalization, and racism within the community.
Poverty	Achieving financial security and reducing poverty for community members.
Violence	Preventing and reducing gender-based violence and violence against women in the community.
Mental health	Promoting positive mental health and well-being for all community members.

To help address these priorities, both municipalities have adopted CityStudio, a transferable, nonprofit, project-oriented, evidence-based model of CCE [36,37]. This model aims to innovate how cities are co-created to become healthier communities and assist community stakeholders in improving their neighborhoods [38]. The model is demand-driven, leveraging expertise and resources from educational institutions to meet the priority needs identified by the city. The CityStudio project cycle encompasses 5 stages, from initial collaboration for project development and confirmation through work on a real site to final project design for scalability [36].

The cities of Ottawa and Thunder Bay have formally launched CityStudio and initiated and completed various projects. To date, 63 projects have been carried out involving community groups, city staff, and faculty and students from 6 academic institutions in these regions. CityStudio Ottawa and CityStudio Thunder Bay are engaged in this research study, providing full access to a list of stakeholders involved in their projects and data on an inventory of CCE projects.

### Study Design

We will apply Mayne’s contribution analysis within a convergent parallel mixed methods design to assess the contribution of CCE to population health in Ottawa and Thunder Bay [39]. A convergent parallel design allows for the simultaneous collection and independent analysis of both qualitative and quantitative data, with results from each strand analyzed for congruence and integration during the interpretation phase [40]. Mayne’s approach is particularly suited for complex interventions such as CCE and has been effectively used in assessing the impacts of interventions and policies in fields such as international development [41] and public administration [42]. However, our recent scoping review suggests that this approach is infrequently applied in health-related interventions [43]. Therefore, our research aims to both generate rigorous evidence of CCE’s impact on health and provide valuable insights for using contribution analysis in health research and evaluation.

The following preliminary evaluative questions will direct our analysis. These questions will be refined further upon the completion of stage 1.

- What are the specific mechanisms and pathways by which CCE influences population health outcomes in Ottawa and Thunder Bay?
- To what extent does CCE contribute to changes in population health outcomes relative to other factors in both cities?

We will specifically use the cause-to-effect strategy, which begins with CCE and works forward to understand how it contributes to health outcomes. The cause-to-effect strategy focuses on identifying “causal packages”—combinations of CCE activities and other contributing factors that collectively explain the observed changes [44]. We will adopt a streamlined version of Mayne’s 6-step approach to contribution analysis, condensing it into the 3 crucial stages described by Delahais [30]. The 3 stages are as follows.

- Stage 1—theory of change development: This stage involves using a 3-phase approach to developing a robust theory of change that accurately represents the potential pathways and external factors describing how CCE is expected to contribute to improved population health outcomes in Ottawa and Thunder Bay. This stage is expected to last 7 months.
- Stage 2—evidence gathering: Both qualitative and quantitative methods are used to generate evidence in line with the established theory of change. The collected evidence forms the basis for empirical examination. This stage is expected to last 11 months.
- Stage 3—theory of change assessment and refinement: This stage involves reviewing the gathered evidence to determine the extent of CCE impacts on population health. Any identified limitations lead to a return to stage 2 for additional data collection until satisfactory evidence is gathered. This stage is expected to last 5 months.

**Figure 1:**
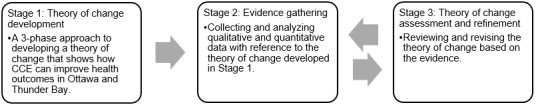
Overview of the research process. CCE: community-campus engagement.

Figure 1 provides a visual overview of the research process, illustrating the dynamic relationship between data collection (stage 2) and theory testing (stage 3) [30].

This study adheres to the Good Reporting of A Mixed Methods Study (GRAMMS) guidelines [45] to ensure comprehensive and transparent reporting of the mixed methods design, including the justification for the approach, the integration of qualitative and quantitative data, and the insights gained from their convergence (Multimedia Appendix 1).

### Stage 1: Theory of Change Development

#### Overview

Stage 1 will use a 3-phase approach to construct a credible theory of change. Each phase corresponds to one of the approaches proposed by Funnell and Rogers [46] for purposeful theory development: (1) deductive, (2) articulation of mental models, and (3) inductive, which will be implemented sequentially in the theory of change development process, with the potential for iteration to accommodate evolving requirements. An overview of each phase and corresponding methods is presented in Table 2. While our approach follows a structured sequence, we acknowledge that the evolving nature of developing a theory of change requires flexibility, allowing for iteration as needed. Each step of the theory of change development will be carefully documented to maintain transparency in this iterative process. This includes tracking the initial assumptions, the evolution of claims based on stakeholder input and empirical evidence, and the methodological adjustments made throughout the process. The documentation will highlight how and why specific claims were refined, discarded, or validated, ensuring that the process remains transparent and aligned with best practices in contribution analysis.

**Table 2:** Overview of the study’s 3-phase approach to developing the theory of change.

Phases	Description	Proposed methods
Phase 1: Deductive	Draft an initial theory of change based on existing theories and knowledge.	Conduct a narrative literature review on the health impacts of CCE^a^, focusing on the 5 shared Ottawa and Thunder Bay priorities.
Phase 2: Mental model articulation	Express the implicit assumptions or mental models of stakeholders.	Hold stakeholder workshops to provide feedback on the drafted theory of change and identify context-specific factors influencing outcomes.
Phase 3: Inductive	Validate and refine the theory of change using practical insights and external expertise.	Conduct semistructured interviews with experts in CCE and program evaluation for validation and refinement.

^a^CCE: community-campus engagement.

#### Phase 1: Deductive

Phase 1 uses Funnell and Rogers’ deductive approach [46] to formulate an initial theory of change based on existing theories and knowledge, ensuring a solid theoretical foundation. To accomplish this, we will conduct a narrative literature review chosen for its flexibility and capacity to identify critical theories, concepts, and findings related to CCE and its impact on population health concerning the five identified areas of population health [47]. The results of this review will lay the groundwork for the development of the theory of change, as detailed in the subsequent sections.

#### Narrative Literature Review Procedures

In collaboration with a librarian, we will design and execute a comprehensive search strategy that encompasses relevant health databases and sources. The selected literature will be imported into Covidence (Veritas Health Innovation Ltd), a web-based literature review tool, for screening by independent reviewers [48]. At least 3 research team members will review various sources, including academic papers, reports, and policy documents, to inform the initial development of the theory of change. Screening criteria will include relevance to the 5 key population health priorities and use for the theory of change.

Team members will create an annotated bibliography for each paper using a standardized template based on Bennett’s hierarchy [49], a framework that categorizes program outcomes (inputs, activities, outputs, and impacts) [50]. The template will be divided into three sections: (1) CCE implementation, focusing on inputs, activities, participation, and reactions; (2) knowledge and practice change, focusing on knowledge, awareness, skills, attitude, and practice change; (3) results and impact, focusing on final health impacts or changes for individuals, communities, systems, or organizations. Team members will hold weekly meetings to share insights from the literature and discuss potential pathways.

#### Development of the Initial Theory of Change

Following the literature review, team members will convene to create the initial theory of change using Vogel’s team-based approach, which is specifically designed to develop theories of change for research projects. This approach includes guiding questions on 5 key areas: context analysis, long-term change, sequence of events, assumptions, and diagram and narrative summary [51]. To document decisions and track modifications, the team will use Theory of Change Online 3.0 (ActKnowledge), a web-based software designed explicitly for the collaborative theory of change building [52]. This initial theory of change will remain adaptable, allowing for iterative revisions based on feedback and new data. Once the initial draft of the theory of change is completed and agreed upon, the team will proceed to the mental model articulation phase.

#### Phase 2: Mental Model Articulation

In phase 2 of the theory of change development, we will use Funnell and Rogers’s mental model articulation approach [46]. This approach will help us integrate stakeholder insights, ensuring they are context-specific, comprehensive, and account for relevant influencing factors. To achieve this, we will conduct participatory workshops with stakeholders involved in CCE through CityStudio Ottawa and Thunder Bay [53,54].

#### Recruitment of Stakeholders

We will use a purposeful sampling strategy to invite stakeholders based on specific criteria such as their location (Ottawa and Thunder Bay), experience with CCE, stakeholder roles (academics, community partners, and city staff), and knowledge related to the 5 shared population health priorities. This approach will ensure a diverse representation of stakeholders, balancing their professional backgrounds, geography, and CCE experience. [55].

Our initial outreach will target CityStudio Ottawa and CityStudio Thunder Bay stakeholders who expressed interest in our workshops during 2 information sessions held in May 2023, which aimed to gather feedback on the proposed protocol and identify potential workshop participants. The participants will include academics, community partners, and city representatives involved in CCE who expressed interest and shared their contact details through a web-based survey during their participation in the information session.

We will engage CityStudio coordinators in Ottawa and Thunder Bay if additional participants are needed. They will circulate an invitation email to their contact lists, which include a broader network of individuals involved in CCE. The email will provide information about the project, the purpose of the workshops, and the importance of their participation. All participants will be asked to sign a consent form before their involvement.

#### Stakeholder Workshops Procedures

We plan to conduct 2 separate workshops conducted via Zoom. The first workshop will assemble 10-12 stakeholders from Ottawa, and the second will gather a similar group from Thunder Bay. Each workshop, lasting 3 hours, will be divided into 2 parts.

- Introduction and overview: We will provide essential details about contribution analysis and the theory of change. We will then present an overview of our draft theory of change and explain the process used to develop it.
- Feedback and identification of context-specific factors: We will use Kranias’ participatory facilitation techniques [56] to collect feedback on the plausibility of our initial theory of change and, for each city, identify the roles of other influencing factors and alternative explanations that could affect the theory of change’s impact pathways. To identify specific contextual factors with participants, we will use the classification of Pawson et al [57], which divides these factors into 4 categories: individual, interpersonal, institutional, and infrastructure. This classification has proven helpful for identifying contextual factors in a workshop setting in previous applications of contribution analysis [50,58]. This classification and stakeholder input will be revisited iteratively as needed, ensuring the theory of change remains aligned with emerging contextual insights.

At least 2 research team members will facilitate each workshop, following a facilitation guide based on Vogel's approach [50]. With participant consent, workshops will be recorded for review purposes. Workshop participants will receive CAD $100 compensation (approximately US $71 at the exchange rate at the time of the study: CAD $1=US $0.71) for their time and contribution and a summary of the key outcomes and changes made to the theory of change for validation.

#### Phase 3: Inductive

Phase 3 involves assessing the theory of change’s coherence, logic, and theoretical foundations and identifying potential gaps or areas for enhancement. Following Funnell and Rogers [46], we will conduct semistructured interviews with experts. This method is particularly effective for gathering in-depth, expert-based insights, which will be instrumental in refining our theory of change and ensuring its robustness and relevance.

#### Recruitment of Experts

We will use a snowball sampling strategy to identify 5-6 experts in population health, CCE, and theory-based evaluation. Our research partners will provide us with a list of potential interviewees. These individuals will receive an invitation via email, including a detailed description of the study and their expected participation. Before participating, they must sign a consent form and declare no conflict of interest. Each expert will be compensated CAD $150 (US $105.87) for their time.

#### Data Collection and Analysis

We will send a documentation package to the experts for review 3 weeks before the interview. This package will include the drafted theory of change and its narrative, the development process used, and an assessment grid based on Mayne’s validated criteria for an in-depth theory of change analysis [31]. The assessment grid will contain questions to assess the overall logic and structure of the theory of change, the clarity of its expected outcomes, the validity of its assumptions, and the independence of assumptions for each causal link [31]. A research team member will contact the experts to ensure they understand the documentation and answer any questions.

The semistructured interviews will combine structured questions from the assessment grid with open-ended questions for comprehensive feedback [53]. To avoid potential bias, the interviews will be conducted by research team members who were not involved in the theory of change development process. During the interview, we will collect their comments and suggestions. All interviews will be recorded for accuracy.

#### Formulation of Contribution Claims

Upon completion of the interviews, we will collate and discuss the differences and similarities between the reviewers’ assessments and the feedback received. Based on their suggestions, we will adjust the theory of change and formulate initial contribution claims. As mentioned, these claims are narrative statements derived from the theory of change that articulates the anticipated pathways through which CCE is expected to improve population health outcomes in the specific contexts of Ottawa and Thunder Bay [30]. The initial theory of change and contribution claims developed during this phase are considered provisional. As new evidence is gathered and stakeholder insights are integrated, these claims will be iteratively refined. This flexibility is essential to capturing the complex and context-specific pathways through which CCE impacts population health. We will proceed to the second stage of the study once we reach a consensus that the theory of change and the generated contribution claims are sufficiently articulated, credible, plausible, and logical.

### Stage 2: Evidence Gathering

#### Overview

In this stage, we will use a mixed methods approach to gather substantial evidence that will be used to examine the theory of change and the associated contribution claims developed in stage 1 [30]. The evidence-gathering process is designed to be adaptive. If emerging data suggest new pathways or causal mechanisms, earlier stages may be revisited, and claims adjusted accordingly. This iterative approach ensures that the contribution claims remain aligned with the evolving evidence base. We will create a data collection plan to identify specific indicators, data sources, and methods for examining each contribution claim and its assumptions. The plan will identify at least three evidence sources for each claim, using Delahais’ triangulation approach [30] for validation, to allow examination from multiple perspectives.

#### Data Collection

We will collect qualitative and quantitative data from various sources: (1) case studies, (2) a web-based stakeholder survey, (3) pathway-specific literature review, and (4) population and community health data. Flexibility in data collection will be maintained by revisiting or expanding data sources as necessary, based on initial analyses or gaps identified during triangulation. Table 3 provides an overview of the data sources and their respective aims.

**Table 3:** Overview of data collection and sources for stage 2.

Data collection	Aim	Data sources
Case studies	Examine the impact of existing CCE^a^ projects with high potential for positive health outcomes in Ottawa and Thunder Bay on the 5 population health outcome areas.	Interviews and document reviews from appropriately 30 CityStudio projects.
CCE Health Impact Survey	Identify the perceived health impacts of CCE projects not selected for case studies.	A web-based survey for stakeholders involved in or impacted by CCE projects through CityStudio Ottawa and CityStudio Thunder Bay.
Pathway-specific literature review	Assess the evidence for CCE’s impact on population health outcomes.	Peer-reviewed research and evaluation literature related to health outcomes in CCE.
Population and community health data	Identify trends in disparities to understand CCE’s impact on health outcomes.	Data from Community Safety and Well-Being Plans of Ottawa and Thunder Bay and other health indicators.

^a^CCE: community-campus engagement.

#### Case Studies

##### Aim

Case studies involve a detailed examination of a selection of CCE projects with the most significant potential to positively influence Ottawa and Thunder Bay health outcomes. They can provide evidence on the theory of change and have often been used in contribution analysis to understand the factors contributing to intervention success or failure in different contexts [46,50].

##### Case Selection

We will collaborate with CityStudio Ottawa and Thunder Bay coordinators to identify and rank approximately 15 CCE projects from each city with the most promise for positively impacting at least one of the shared population health priorities. We aim to have at least 1 case study for the 5 health priorities. We will create a checklist based on a review of existing tools and measurement instruments to evaluate projects on successful engagement, possible health impact, and data reliability and accessibility. The final selection of projects will be determined through group consensus using the checklist. The rationale for each selection will be documented in a report for transparency and accountability. The case study pool may be expanded or adjusted accordingly if new CCE projects emerge during data collection that shows high potential for positive outcomes.

##### Data Collection Tools for Case Studies

As suggested by Yin [59], we will use 2 sources to enhance the rigor of case studies: semistructured interviews and organizational documents.

#### Semistructured Interviews

We will conduct semistructured 1-hour interviews with key stakeholders in each selected case using an interview guide based on the components of our theory of change. Each interview will focus on uncovering the initiative’s perceived impacts, foundational mechanisms, and broader outcomes. We will record each interview and take notes during the interviews. Participants will be asked to sign a consent form before the interview and receive CAD $25 (US $17.64) compensation for their contribution. The interviews will be transcribed, with copies uploaded to a secure database in PDF format.

#### Project Document Review

Our study team will review documents related to selected CityStudio projects, such as project descriptions, monitoring and evaluation reports, output reports, progress reports, and annual reports. We will obtain project documentation from our research collaborators, CityStudio Ottawa and CityStudio Thunder Bay. This review will help us identify expected activities, outputs, and outcomes and provide insights into the strategies used, challenges encountered, and the overall progress and impact of the projects. The total number of documents we will review will depend on the number of projects and their complexity. However, we aim to review as many relevant documents as possible to understand the projects comprehensively. All project documentation will be securely stored for future reference and analysis.

#### CCE Health Impact Survey

##### Aim

The CCE Health Impact Survey is designed to collect insights from diverse stakeholders involved in or impacted by CCE projects in Ottawa and Thunder Bay. The survey aims to understand potential health outcomes related to housing, discrimination, poverty, violence, and mental health.

##### Sampling Strategy

We plan to gather data from individuals who have actively participated in or been affected by projects from CityStudio Ottawa or CityStudio Thunder Bay. Individuals must not be case study participants to be eligible for the survey. They must also belong to one of the following stakeholder groups: students, faculty or researchers, city representatives, or community members. If survey participation is lower than expected or new stakeholder groups are identified, the sampling strategy may be adjusted to ensure comprehensive data collection.

Potential participants will be selected for our survey through convenience sampling. The CityStudio coordinators from Ottawa and Thunder Bay will email these individuals, outlining the project’s objective and emphasizing the importance of their participation. Before participating in the survey, individuals must complete a consent form.

The recruitment for the survey will conclude once we have received a sufficient number of completed surveys to ensure robust statistical analysis. The exact number will be determined using the Cochran formula [60], which accounts for a 95% CI and a 5% margin of error. Additionally, we will aim to ensure a diverse and representative sample by including participation from each stakeholder group and maintaining balanced representation from both cities.

##### Measures

The survey will consist of 3 sections. The first section will collect details about the respondents’ involvement in the CCE project, such as their role, duration of participation, and affiliation with the organization. The second section will delve into the specifics of the CCE project, including its objectives, the strategies used for its implementation, the deliverables, and the stakeholders involved. The final section will focus on the perceived health impacts of the CCE project. Respondents will be asked to share their observations and experiences and to report any noticeable changes they have observed in 5 key areas: housing, discrimination, poverty, violence, and mental health.

Before launching the survey, we will conduct a pretest with potential participants from all targeted groups to ensure its clarity and relevance. The survey will include Likert scales and open-ended questions, and we anticipate it will take approximately 15 minutes to complete. Participants will receive a CAD $25 (US $17.64) compensation upon completion.

#### Pathway-Specific Literature Review

##### Aim

We will synthesize peer-reviewed literature to evaluate the significance and evidence level for each pathway in the theory of change. This will help us understand how these pathways influence the impact of CCE on population health outcomes. Our process will include an analysis of studies that have investigated similar pathways. We will scrutinize their methodologies, findings, and conclusions and assess the overall robustness and limitations of the existing literature. The literature review process will be iterative, allowing for additional rounds of review if new gaps or needs are identified during data collection or analysis. The review procedure for pathway-specific literature will adhere to the guidelines for the narrative literature review outlined in stage 1.

#### Population and Community Health Data

##### Aim

We will collect health data to evaluate the impact of CCE on health outcomes in Ottawa and Thunder Bay. This will include information on health behaviors, outcomes, access to health care services, and social determinants of health. By examining population characteristics, health measures, and factors influencing health outcomes, we aim to understand how CCE affects immediate and downstream health outcomes. We will also draw on indicators, results, and performance measures developed and tracked by the municipal CSWBP teams. Domains of eligible indicators include (1) socioeconomic environment (income, social support, education, and employment); (2) physical environment (green space, air quality, housing, and transportation); (3) healthy child development (birth weight, immunization, and early development indicators); (4) health behaviors (smoking, substance use, diet, and physical activity); (5) individual capacity and coping skills; (6) biology and genetic endowment; (7) health services; and (8) sociodemographic environment (culture, race or ethnicity, and sex or gender).

#### Data Analysis Approach

Our data analysis approach involves qualitative and quantitative analysis, followed by aggregation.

#### Qualitative Data Analysis

At least 2 research team members will perform a thematic analysis of project documents and semistructured interviews. We will adhere to the 6-step thematic analysis coding framework proposed by Braun and Clarke [61], which includes (1) familiarization of data, (2) generation of codes, (3) combining codes into themes, (4) reviewing themes, (5) determining the significance of themes, and (6) reporting of findings [57]. The thematic analysis will be conducted iteratively, allowing for additional rounds of coding and theme development if new insights or patterns emerge. For example, if initial analysis reveals unexpected causal pathways or unanticipated factors, further in-depth analysis will be conducted to explore these themes. We will focus on identifying linguistic indicators of change or cause-effect relationships in interviews using Nour et al's argumentative discourse analysis [62]. This process entails a detailed examination of specific linguistic markers such as “it is obvious/clear that” and “compared to.” These markers serve as indicators of patterns or relationships in the data. We will use NVivo software (Lumivero) [63] for data organization and analysis.

#### Quantitative Data Analysis

We will collect quantitative data from CityStudio projects, the CCE Health Impact Survey, and existing, deidentified publicly available population and community health indicators. We will use descriptive and appropriate inferential statistical analysis using statistical software (STATA version 14.2, StataCorp LLC) [64] to describe and analyze patterns, relationships, and impacts pertinent to the contribution pathway.

#### Aggregation of Evidence

We will use Delahais and Toulemonde's Evidence Analysis Database [32] to combine, examine, and summarize the evidence gathered for our theory of change and the contribution claims and related assumptions or risks associated with it. Triangulation will be conducted iteratively, allowing for the inclusion of new evidence sources or reexamination of previously collected data if discrepancies are identified during the analysis. The database will be structured as a digital spreadsheet, with each row corresponding to a piece of evidence linked to a specific contribution claim from our theory of change. For each piece of evidence, we will record the following details: label (an identifier for the evidence), statement (a brief description of the evidence), data source (case studies, CCE Health Impact Survey, pathway-specific literature review, and population and community health data), type of evidence (primary or secondary), interpretation (confirming or refuting the claim), strength of evidence (rating of the evidence’s strength and justification), and comments (additional notes or observations about the evidence or implications based on the city).

### Stage 3: Theory of Change Assessment and Refinement

#### Overview

In this stage, our objective is to assess and refine the contribution claims formulated from our theory of change based on the evidence gathered and analyzed in the preceding stage. To ensure a rigorous assessment, we will adopt Downe's approach [65] of using an independent review panel, consisting of experts recruited in the inductive phase of the theory of change development (stage 1). If those experts are unavailable, we will use a snowballing strategy to find other relevant experts.

#### Review Panel Procedures

We will send a review package to the experts 3 weeks before the panel meeting. This package will include the draft theory of change and its narrative, the development process used, and an assessment grid for the contribution claims. The number of claims will be determined based on the theory of change. The assessment grid will contain questions to assess the plausibility and strengths of these claims, along with the overall logic and structure of the theory of change. A research team member will contact the experts to ensure they understand the documentation and answer any questions they may have. Workshop participants will be asked to sign a consent form and will receive compensation of CAD $200 (US $141.15) for their time.

The panel meeting will last 4 hours and will be facilitated by 2 research team members who were not involved in data collection and analysis. It will be divided into 2 parts.

- Introduction and question-and-answer: Panel members will receive presentations from the research team on the methods and contribution claims, followed by a question-and-answer session where they can ask for clarifications or additional information about the presented materials.
- Assessment: Panel members will be invited to share their feedback on the contribution claims and rate them by consensus according to their plausibility and rigor on a scale from 1=very weak to 4=very strong. They will use the aggregated data in Delahais and Toulemonde's Evidence Analysis Database [32] to verify the coherence of the contribution claims by contrasting both qualitative and quantitative data collected on them. Contribution claims with low scores will be used to prompt panel members for additional information sources. This feedback leads to another round of targeted data collection using data sources described in phase 2, ensuring our findings are reliable, accurate, and context specific.

#### Refinement and Reporting

After the panel meeting, the research team will compile a detailed report that includes the theory of change, the supporting evidence, and the panel’s ratings and feedback. By documenting the evidence and the narrative of how it substantiates the theory of change, along with the expert panel’s evaluations, this report aims to present a comprehensive and credible account of the contribution story. The report will also highlight the iterative nature of the process, documenting how contribution claims were progressively refined, validated, or adjusted based on new evidence. This transparency in the evolution of claims is crucial for demonstrating the credibility and robustness of the evaluation findings.

### Ethical Considerations

This study adheres to the ethical guidelines and principles relevant to human research. It has been granted ethical approval by the Bruyère Research Ethics Board (M16-23-009) on May 4, 2023. Ethical approval for our site in Thunder Bay is currently pending, and as such, no data will be collected at the site until we receive the necessary approval. This research is supported by the Canadian Institutes of Health Research (PJT-180529). All participants will provide written informed consent after being fully informed of the study's purpose, procedures, potential risks, and their right to withdraw at any time without consequence. Identifiable information will not be disclosed in study publications or presentations. Our approach to compensation is described in the Method section.

## Results

Our study is in the second stage, focusing on evidence gathering. We anticipate study results will be available by the end of May 2026. The findings will provide insights into the impact of CCE initiatives on population health in Ottawa and Thunder Bay, contributing to the broader understanding of these complex relationships.

## Discussion

### Expected Findings

CCE is increasingly recognized for its potential to address local health needs. However, a more rigorous and precise understanding of its impact on population health is needed. This research uses a 3-stage, convergent parallel mixed method design combined with contribution analysis to provide robust evidence on how CCE has improved population health in Ottawa and Thunder Bay, 2 distinct settings in Ontario, Canada. The study will provide evidence for CCE's impact on 5 shared population health priorities for both municipalities: housing, discrimination, poverty, violence, and mental health. By focusing on reducing uncertainty and disentangling the complex interactions among contributing factors, our study will provide a clearer and more nuanced understanding of how and under what conditions CCE contributes to these health outcomes. This approach goes beyond simply assessing whether CCE has an impact and delves into the mechanisms, contexts, and pathways through which these contributions occur.

While Mayne’s approach has been effectively used in assessing the impacts of interventions and policies in fields such as international development [32] and public administration [39], its application in health research is less explored. Our study aims to generate rigorous evidence of CCE’s impact on health and provide valuable insights for applying contribution analysis in health research and evaluation. Our protocol ensures robust findings using 3 triangulation types: data triangulation using a mixed methods approach, analyst triangulation by involving various members of the research team and stakeholders in all 3 stages of the study, and external validation for an objective evaluation of our theory of change and contribution story. This ensures reliable and context-specific results.

The implications of these findings are substantial. Policy makers will be equipped with evidence-based insights that can be used to refine community engagement strategies and optimize resource allocation for improved population health outcomes. Health education institutions can leverage these insights to strengthen their community engagement endeavors, enrich curricula, and demonstrate their commitment to social accountability. Furthermore, communities will gain invaluable evidence for a scalable CCE model that can be applied across diverse settings.

While this study focuses on CCE initiatives in Ottawa and Thunder Bay, the findings are intended to be transferable to other contexts. By identifying common configurations, mechanisms, and contextual factors that contribute to health outcomes, this study offers insights that can be adapted and tested in different settings. For instance, understanding the conditions under which CCE initiatives are most effective can inform similar efforts in municipalities with comparable socioeconomic conditions or health challenges. The transferability of findings will be discussed in detail, emphasizing the specific aspects of the intervention that may be generalizable across contexts.

### Limitations

The precision of our analyses may be constrained by the challenge of obtaining high-quality data for CCE projects. Contribution analysis is relatively novel, adding complexity to our study. Stakeholder participation in participatory research presents challenges such as ensuring effective communication, equitable authorship access, and fair compensation mechanisms. The potential for participant bias exists, which could affect data objectivity but will be mitigated by maintaining neutrality during data collection and analysis and providing clear study guidelines to participants. Another limitation is the dependence on perceived outcomes and correlational relationships, which will be addressed through triangulation methods involving multiple data sources to cross-verify findings and provide a comprehensive understanding of impacts.

### Conclusions

This protocol outlines a novel approach to assessing the impact of CCE on Ottawa and Thunder Bay population health. The study aims to provide robust evidence on how CCE contributes to population health improvements by using a convergent parallel mixed methods design and contribution analysis. The study’s findings are expected to fill critical knowledge gaps regarding the specific pathways, configurations, and contextual factors that influence CCEs. These insights will be valuable for policy makers, health education institutions, and communities seeking to optimize their engagement strategies. Despite the limitations, such as geographical scope and potential participant bias, the iterative, flexible, and rigorous design enhances the reliability and applicability of the findings. The research team anticipates that the findings will validate and refine the contribution claims derived from the theory of change and guide future research and interventions in community engagement and health. This study represents a significant step forward in understanding and harnessing the potential of CCE in improving population health.

## Appendix

Multimedia Appendix 1 Good Reporting of a Mixed Methods Study (GRAMMS) checklist.

## Abbreviations

CCE: community-campus engagement
CSWBP: Community Safety and Well-Being Plans
GRAMMS: Good Reporting of A Mixed Methods Study
