# Supplementary material for: Evaluating the Impacts of Community-Campus Engagement on Population Health in Ottawa and Thunder Bay, Canada: Protocol for a Mixed Methods Contribution Analysis
Source: JMIR Res Protoc. 2025 Jan 17;14:e58546. doi: 10.2196/58546 (PMC11786136; doi:10.2196/58546)
Supplement: Multimedia Appendix 1 [file resprot_v14i1e58546_app1.docx]

**Table S1.** Good Reporting of a Mixed Methods Study (GRAMMS) checklist.

| Guideline | Section | Page(s) |
| --- | --- | --- |
| 1. Describe the justification for using a mixed methods approach to the research question | Methods - Procedures | Page 8 |
| 2. Describe the design in terms of the purpose, priority, and sequence of methods | Methods - Procedures | Pages 7-8 |
| 3. Describe each method in terms of sampling, data collection, and analysis | Procedures | Pages 7-8 |
| 4. Describe where integration has occurred, how it has occurred, and who has participated in it | Design | Pages 7-8 |
| 5. Describe any limitation of one method associated with the presence of the other method | Discussion | Pages 15-17 |
| 6. Describe any insights gained from mixing or integrating methods | Discussion | Pages 15-17 |

### **Explanation for Each Checklist Item**

1. Justification for Mixed Methods: In the “Methods” section under procedures (Page 8), the manuscript explains why a mixed-methods approach is necessary to address the complexity of evaluating CCE’s contribution to health outcomes.
2. Design Description: The design is outlined in the “Methods” section, with details on the purpose, priority given to qualitative and quantitative methods, and how they are sequenced (Pages 7-8).
3. Sampling, Data Collection, and Analysis: The “Procedures” section (Pages 7-8) and the “Analysis” section (Pages 9-10) provide specific details on sampling strategies, data collection procedures, and analysis techniques for both qualitative and quantitative methods.
4. Integration: The manuscript discusses where and how integration occurs, along with who participates in this process, in the “Design” section (Pages 7-8).
5. Method Limitations: Any limitations specific to the presence of both qualitative and quantitative methods are discussed in the “Discussion” section (Pages 15-17).
6. Insights from Integration: The “Discussion” section (Pages 15-17) highlights key insights gained from integrating the methods, such as enhanced understanding of the causal pathways and better contextualization of quantitative findings.
